# Rhythmic TMS as a Feasible Tool to Uncover the Oscillatory Signatures of Audiovisual Integration

**DOI:** 10.3390/biomedicines11061746

**Published:** 2023-06-17

**Authors:** Riccardo Bertaccini, Giuseppe Ippolito, Luca Tarasi, Agnese Zazio, Antonietta Stango, Marta Bortoletto, Vincenzo Romei

**Affiliations:** 1 Centro Studi e Ricerche in Neuroscienze Cognitive, Dipartimento di Psicologia, Alma Mater Studiorum—Università di Bologna, 47521 Cesena, Italy; riccardo.bertaccini5@unibo.it (R.B.); giuseppe.ippolito4@studio.unibo.it (G.I.); luca.tarasi2@unibo.it (L.T.); 2 Neurophysiology Lab., IRCCS Istituto Centro San Giovanni di Dio Fatebenefratelli, 25125 Brescia, Italy; agnese.zazio@cognitiveneuroscience.it (A.Z.); antonietta.stango@cognitiveneuroscience.it (A.S.); marta.bortoletto@cognitiveneuroscience.it (M.B.); 3 Laboratory of Cognitive Neuroscience, Department of Languages and Literatures, Communication, Education and Society, University of Udine, 33100 Udine, Italy; 4 Facultad de Lenguas y Educación, Universidad Antonio de Nebrija, 28015 Madrid, Spain

**Keywords:** sound-induced flash illusion, rhythmic transcranial magnetic stimulation, electroencephalography, stimulus onset asynchrony, individual alpha frequency, alpha oscillations, cross-modal illusions

## Abstract

Multisensory integration is quintessential to adaptive behavior, with clinical populations showing significant impairments in this domain, most notably hallucinatory reports. Interestingly, altered cross-modal interactions have also been reported in healthy individuals when engaged in tasks such as the Sound-Induced Flash-Illusion (SIFI). The temporal dynamics of the SIFI have been recently tied to the speed of occipital alpha rhythms (IAF), with faster oscillations entailing reduced temporal windows within which the illusion is experienced. In this regard, entrainment-based protocols have not yet implemented rhythmic transcranial magnetic stimulation (rhTMS) to causally test for this relationship. It thus remains to be evaluated whether rhTMS-induced acoustic and somatosensory sensations may not specifically interfere with the illusion. Here, we addressed this issue by asking 27 volunteers to perform a SIFI paradigm under different Sham and active rhTMS protocols, delivered over the occipital pole at the IAF. Although TMS has been proven to act upon brain tissues excitability, results show that the SIFI occurred for both Sham and active rhTMS, with the illusory rate not being significantly different between baseline and stimulation conditions. This aligns with the discrete sampling hypothesis, for which alpha amplitude modulation, known to reflect changes in cortical excitability, should not account for changes in the illusory rate. Moreover, these findings highlight the viability of rhTMS-based interventions as a means to probe the neuroelectric signatures of illusory and hallucinatory audiovisual experiences, in healthy and neuropsychiatric populations.

## 1. Introduction

How human perceptual systems handle signals gathered by our sensorium is pivotal to adaptive behavior. Relatedly, multisensory integration is perhaps the most demanding among the environmental challenges perception has to cope with [1,2]. Bayesian theories posit that two main questions should be addressed for observers to efficiently accommodate modality-diverging inputs into a meaningful percept. First, they must ascertain whether such inputs share a common source [3,4]. If so, they then need to evaluate how reliable they are in sensory terms [5,6]. Any failure to resolve either of these problems is likely to prompt misperceptions, fueling maladaptive responses and, in the long run, abnormal behavior [2,7]. 

To date, translational evidence is suggestive of far-reaching deficits in cross-modal processing across clinical phenotypes such as autism [8], dyslexia [9], and schizophrenia [10], in which hallucinations are the first-rank symptom. However, as novel biobehavioral models of perceptual decision-making emphasize [11], episodes of disrupted multisensory integration could be witnessed in healthy subjects as well. It is the case of cross-modal illusions, where the simultaneous (or near-simultaneous) occurrence of stimuli differing in modality misleads the observer either into perceiving a non-existing phenomenon or not perceiving an existing one. Noteworthy examples are the McGurk effect [12], the Rubber-Hand illusion [13], and the Ventriloquist effect [14], which have been construed as a by-product of a sensory system overruling the other one it is interacting with. Along this line of the literature lies in what has also been referred to as the Sound-Induced Flash Illusion (SIFI, [15]).

In the SIFI, the presentation of a single flashing dot coupled with temporally close pairs of acoustic stimuli has been found to drive observers into reporting a second, illusory flash. As clarified by additional inquiries on the matter, the closer in time acoustic inputs occur, the likelier the SIFI (and vice versa) [16,17]. These dynamics subsume the broader functional architecture of cross-modal interplay, in which the sensory primacy of audition over vision has been thought to shape the latter’s temporal binding windows (TBW). Cortical spots are most likely to host these kinds of operations have been proven to lie on both the occipital and temporal lobes, and the pathways in between [18,19]. The former hosts the primary visual cortex, whose hemodynamic responses and structural integrity have been shown to predict SIFI proneness [20,21]. Furthermore, anatomical evidence hints at a rich network of synaptic afferents branching out from primary auditory cortices to the occipital pole [22,23]. Under this perspective, when temporal neurons fire in response to an acoustic stimulation (i.e., beeps), the resulting signals were shown to dictate windows of occipital excitability (i.e., TBWs) phase-locked at 10 Hz [24,25,26].

Cognitive electrophysiology via electroencephalography (EEG) has been thus embraced to better detail the oscillatory underpinnings of this phenomenon, with a specific focus on posterior alpha rhythms (8–12 Hz). Away from cortical idling, phasic fluctuations within this frequency band have been proven to index cortical excitability [27,28,29], and deploy attentional resources via sensory gating [30,31], and route in a feedback fashion top-down predictions along the cortical hierarchy (i.e., predictive coding [32,33,34,35]). Alpha oscillations have also been assumed to scaffold perception with a temporal frame, parsing the ever-flowing stream of visual events into discrete epochs by virtue of their cycling rate [36,37]. As principled by the discrete sampling hypothesis account, the faster (or slower) these rhythms oscillate, the higher (or lower) the observers’ visual sampling rate and their perceptual or cognitive accuracy [38,39,40] (but see [41] for a different perspective).

Altogether, these assumptions are of uttermost relevance to the understanding of the SIFI. Indeed, the likelihood of reporting an illusory percept during SIFI paradigms was found to dramatically increase (vs. decrease) for stimulus onset asynchronies (SOAs, i.e., the temporal spacing between auditory inputs within each acoustic pair, roughly corresponding to the inter-beep interval) lower (vs. higher) than 100 ms (i.e., the average length of an alpha cycle) [42]. Spectral inquiries informed by such pieces of evidence substantiate a mechanistic scenario wherein specific pre-stimulus alpha features seem to be predictive of SIFI outcomes [43]. In particular, the speed at which posterior alpha waves resonate (i.e., IAF, defined as the local power spectrum peak within the frequency band spanning from 7 to 13 Hz [44]) was shown to be intertwined with SIFI-related performance, with faster rhythms entailing a finer sampling rate, and reduced temporal windows wherein the illusion is experienced [42,45,46].

The case for a causative contribution of alpha waves to the SIFI was further bolstered via transcranial alternating-current stimulation (tACS). When delivered at the proper frequency rate, tACS ignites a physiological response known as “entrainment” (i.e., fine-tuning endogenous neuronal firing to a frequency imposed by an external oscillator) [47,48,49,50]. Crucially, entrainment allows for amplitude modulation and to either up- or down-shift oscillatory peak frequencies toward faster or slower cycling rates [51,52]. Concerning the SIFI, entrainment-based paradigms confirmed that pre-stimulus alpha rhythms orchestrate multisensory synergisms by dictating the observer’s perceptual sampling rate via their frequency peak [42]. However, no actual evidence linking the behavioral readouts of this illusory phenomenon with tACS-driven changes to IAF’s speed has been gathered so far, as it is still a matter of lively debate whether any online EEG recording is viable when delivering such protocol [53,54].

Of note, slower IAFs coupled with higher proneness to the SIFI (i.e., wider TBWs) have been recursively found in individuals lying along the schizophrenic continuum [55,56,57,58], with the degree of impairment in both domains proportionate to the severity of positive symptomatology. These findings reinvigorate the translational virtue of combining cross-modal tasks, such as the SIFI, with electrophysiology and neurostimulation. A promising tool that might serve this purpose is transcranial magnetic stimulation (TMS). 

TMS-based protocols entail the release of transient magnetic bursts over a distinct scalp region, which perturb the electric homeostasis of the cortical mantle lying underneath [59]. In addition to being a safe and non-invasive technique, TMS has been shown to be an effective supplement for tACS [60,61]. This approach harnesses the entraining nature of rhythmic-TMS (rhTMS) and has often been capitalized upon the same way as tACS [62,63,64,65,66], and paired with online EEG recordings. As of today, combined rhTMS-EEG was used to uncover what resonating frequencies are best suited for, respectively, optimal propagation of motoric signals [67], auditory working memory performance [68], and visual detection [69,70]. With regard to the latter, causal evidence for the mechanistic contribution of alpha waves to visual sampling (i.e., discrete sampling hypothesis) has recently been provided through TMS-driven rhythmic entrainment [38]. All in all, rhTMS appears to be endowed with many convenient features for charting the oscillatory mechanics that govern perception, multisensory integration, and, more narrowly, the SIFI. 

However, two main issues hinder the implementation of rhTMS routines in audiovisual tasks, and both concern the physics relative to TMS. First, each magnetic pulse the machine emits is concurrent with a loud clicking noise, which might peak up to 80 decibels. As audiovisual tasks, such as the SIFI, revolve around illusory visual experiences conveyed by timed acoustic stimuli, a crucial question to be addressed is whether TMS-associated clicks impact or not the perceiver’s proneness to double-flash reports, either by enhancing or most likely disrupting the illusory percept. Relatedly, every magnetic pulse elicits cutaneous sensations and muscle twitches over the scalp. Given the impact somatosensations have on the illusion itself [71,72,73], it remains to be tested the degree to which rhTMS interacts or not with the SIFI, prompting unintended changes to the illusory rate. 

In light of these considerations, the translational meaning of the SIFI as a proxy of multisensory deficits along the schizotypal continuum, the orchestrating role ascribed to posterior alpha rhythms in regard, and the potential rhTMS-based investigations retain to explore such a relationship; thus, we seek to clarify whether some preconditions fundamental to the adoption of this protocol are met. Namely, we want to ascertain whether TMS-induced clicking noise and cutaneous sensations might somehow perturb the behavioral readouts of the SIFI. We did so by tracking accuracy rates (i.e., inverse proneness to the illusion) of healthy individuals asked to perform a SIFI paradigm under different temporal conditions, while Sham and rhTMS were delivered (in a block-wise fashion) to the occipital pole at frequency rates matching the IAF, during the pre-stimulus time window.

Importantly, as long as each of the aforementioned stimulation conditions are proven to exert no influence on the occurrence of the illusion, robust evidence will be provided for the feasibility of rhTMS as a tool to gather clinical insights into the oscillatory markers behind multisensory impairments witnessed in individuals suffering from schizophrenia.

## 2. Materials and Methods

### 2.1. Participants

All the experimental sessions were carried out at the Center for Studies and Research in Cognitive Neuroscience in Cesena (Italy). A total of 27 healthy and right-handed volunteers (22 females, mean age 23.11 ± 2.06 s.d. years old), naive as to the purpose of the study, were recruited from a broader population of individuals who already participated in EEG studies performed at our laboratory between the end of 2022 and the beginning of 2023. Written informed consent was obtained from all the participants prior to the experimental testing, which was conducted in accordance with the Declaration of Helsinki and approved by the Bioethics Committee of the University of Bologna (Prot. 140758, 9 October 2018). All participants reported normal or corrected-to-normal vision, no history of psychiatric or neurological disorder, and no counterindication with respect to TMS protocols. No monetary compensation was provided for those who took part in the study.

### 2.2. Procedure

Each experimental session had a duration of approximately 70 min and progressed as follows: Upon signing the informed consent, participants were seated on a chair 57 cm distant from a 17” CRT monitor (refresh rate 60 Hz, 1280 × 1024 resolution) in a dim-lighted and sound-attenuated room. An electrodeless EEG cap, arranged according to the 10–10 international system (i.e., 64 electrode spots), was fitted in order to pinpoint the location over the scalp (i.e., the occipital pole, electrode Oz) to be targeted via rhTMS. 

After receiving detailed instructions concerning the task, participants performed five interleaved blocks of a within-subjects SIFI paradigm. As the literature shows [15], the SIFI entails the report of a second (i.e., illusory) visual percept when a single flashing dot is coupled with pairs of interleaved acoustic stimuli. Such effect is reliant on the temporal spacing between auditory inputs (i.e., SOAs), whose length has been proven to modulate the propensity to experience the illusion [15,42]. On average, SOAs shorter vs. longer than 100 ms have been associated with an increase vs. decrease in the proneness to the SIFI. As such, the SIFI task herein employed was characterized by three distinct SOAs (i.e., 50, 100, and 150 ms), presented in a randomized order across trials within each block. This allowed us to cover, respectively, three different behavioral scenarios: higher (i.e., 50 ms SOA), medium (i.e., 100 ms SOA), and lower (i.e., 150 ms SOA) likelihood of a double-flash report. 

**Figure 1:**
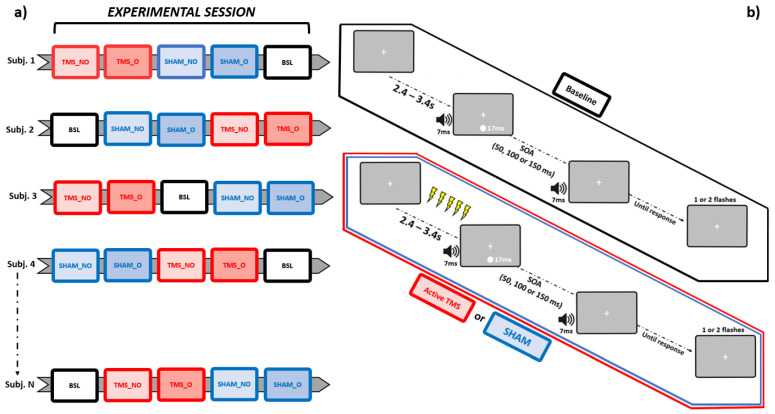
Schematic representation of, respectively, the pseudorandomized-counterbalanced design adopted for this study (leftmost panel, **a**), and the trial structures relative to the SIFI task, sorted as a function of each stimulation condition (rightmost panel, **b**). BSL = Baseline, SHAM_NO = non-overlapping Sham, SHAM_O = overlapping Sham, TMS_NO = non-overlapping TMS, TMS_O = overlapping TMS.

Importantly, each block differed in terms of stimulation conditions from all others. Participants completed a Baseline block (no stimulation provided), along with Sham and active rhTMS, both administered according to two different protocols: “overlapping” and “non-overlapping” (see Section 2.5 below), where the occipital pole was stimulated with an output frequency matching the IAF. This was done following a pseudorandomized counterbalanced order across individuals. That is, the Baseline block could occur, respectively, at the beginning, in the middle, or at the end of each experimental session. Likewise, the order of Sham and active rhTMS blocks was pseudorandomized following a counterbalanced design across subjects, which, however, was implemented independently from that adopted for the Baseline blocks (Figure 1a). This allowed to prevent any practice-induced bias from differentially perturbing behavioral measurements relative to a specific condition. 

### 2.3. SIFI Task

The SIFI was run on a MatLab-equipped (version 2015b, MathWorks) Windows machine via Psychophysics Toolbox (version 3.0). Each trial started with a fixation cross presented on a grey background at the center of the screen. After a random time interval spanning from 2400 to 3400 ms, a single visual stimulus (white-colored circle subtending 2 degrees of visual angles and 5 degrees of visual angles below the fixation cross) was presented for the duration of a refresh rate (~17 ms for a 60 Hz monitor), simultaneously with a 7 ms-long acoustic stimulus (~88 db sinusoidal tone at 3500 KHz, sampling rate 44.1 KHz) emitted from a pair of stereo PC speakers placed at both sides of the screen. After an SOA of either 50, 100, or 150 ms, a second 7 ms-long acoustic stimulus was emitted. At the end of each trial, participants were asked to report (by pressing a key button with their right hand) whether they saw 1 vs. 2 flashes. Each trial’s response type was stored for the subsequent statistical analyses. Let it be further highlighted that what resulted from this procedure was that, in every trial within each experimental session, one flash only was actually presented. As such, the occurrence of 2-flashes reports was to be ascribed to the illusory drive exerted by the SIFI itself.

Every block consisted of 75 trials, equally distributed according to the three aforementioned SOAs (i.e., 25 trials per each SOA, presented in a randomized order), for a total of 375 trials per experimental session. During those experimental blocks (4 out of 5) in which rhTMS (either Sham or active) was delivered, magnetic trains were discharged within the pre-stimulus time window. Namely, the temporal span immediately prior to the onset of the audiovisual pair (Figure 1b).

### 2.4. EEG Recordings 

To control for the impact of both the Sham and active rhTMS protocols on the perceived illusion, participants’ IAFs (as assessed from electrode Oz) were computed by resorting to resting state EEG data (rsEEG) previously recorded at our lab. According to the sampling rate hypothesis, stimulation at the IAF should not have any impact on the illusion rate, so any modulation of the SIFI should be of non-cortical origin and, therefore, similarly observed for both the Sham and active stimulation.

EEG data were recorded at rest while participants were seated on comfortable chairs in a dim-lighted and sound-attenuated room. rsEEG data had a varying duration spanning from 2 to five 5 min and were recorded in the eyes-open condition (i.e., participants were asked to continuously stare at a fixation cross presented on a screen), using 64 active Ag/AgCl electrodes cap (ActiChamp, Brain Products, Gilching, Germany), arranged according to the 10–10 international system. The software Brain Vision Recorder (Brain Products Italia Srl, Putignano, Italy) was used to record the electroencephalogram at 1000 Hz at each electrode. 

Electrodes were online referenced to the FCz electrode, and impedance levels were kept below 10 kΩ. After the impedance check and prior to the recording, the EEG trace was visually inspected to make sure no artifacts from non-physiological sources (e.g., power lines noise, bad electrode contact) were not contaminating the signal. This check was reiterated while the rsEEG recording was ongoing, with a particular focus on occipital channels. 

### 2.5. Rhythmic Transcranial Magnetic Stimulation (rhTMS)

Magnetic pulsations discharged during both the Sham and active rhTMS blocks were generated by a TMS machine (TMS Rapid, Magstim Company, Whitland, UK) and administered via a 70 mm figure-of-eight coil. Five interleaved biphasic pulses (maximum field strength ~1.55 T) were delivered over the cortical spot corresponding to the Oz electrode (as localized using the electrodeless EEG cap participants had to wear) during each trial’s pre-stimulus time window. 

**Figure 2:**
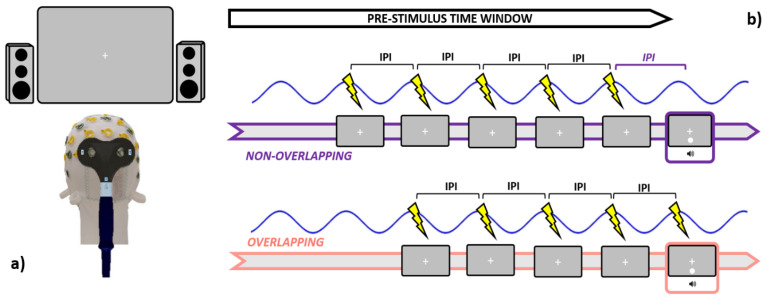
Graphical summary is relative to the stimulation specifics. Leftmost panel (**a**): participants were seated 57 cm distant from the screen, while active TMS was delivered to the occipital pole (electrode Oz) with the coil held tangentially to the scalp. Rightmost panel (**b**): during the pre-stimulus time window, both Sham and Active TMS were delivered right before the occurrence of the audiovisual pair. In the non-overlapping conditions, the last pulse within each train was administered an IPI prior to the audiovisual onset, while in the overlapping conditions, the last pulse was emitted simultaneously with the audiovisual pair. IPI = inter-pulse interval (1000/IAF).

The coil was held tangentially to the scalp, with an orientation set to induce currents running in a posterior-anterior direction during active rhTMS blocks (Figure 2a). Conversely, Sham stimulation was performed by holding the coil orthogonally to the scalp. This latter orientation allowed us to keep the source from which pulses were emitted (and thus the clicking noise) as close as it was when delivering active rhTMS whilst preventing the stimulation from being actually deployed. Maximal stimulator output (MSO) was set at 57%, an intensity level near to those associated with induction of visual phosphenes when TMS is delivered to occipital loci [62,74]. 

TMS was administered in a rhythmic fashion by pacing the release of magnetic bouts according to the IAF. In brief, the inter-pulse interval (IPI, estimated in ms) within each train was set in order to mimic the speed at which the individual’s alpha waves intrinsically oscillate (IPI = 1000/ IAF). Crucially, both Sham and active rhTMS have been delivered following two different timing protocols. In the “overlapping” blocks (e.g., SHAM_O and TMS_O), every train was discharged such that the last magnetic bout within was released in overlap with the presentation of the audiovisual pair, in line with most of the literature employing entrainment protocols [38,62,65,67,68,69,70,75]. On the other hand, in the “non-overlapping” blocks (e.g., SHAM_NO and TMS_NO), the last magnetic pulse in each train was transmitted an IPI prior to the presentation of the audiovisual pair (Figure 2b). Since rhTMS-induced entrainment after-effects are rather short-lived [60], this approach enabled us to disentangle whether trains delivered in overlap with the audiovisual pair (i.e., ensuring the soundest entrainment) might perturb or suppress the SIFI, as compared to “non-overlapping” trains. Germane to this, as our focus was also to clarify to which degree TMS-generated clicking noise might interact with the perception of task-related acoustic stimuli, we quantified the loudness of rhTMS clicks via a digital phonometer. This operation returned an intensity value of around 78 decibels, which prompted us to set the output of both the PC speakers at a slightly higher level (88 decibels, see Section 2.3). Timing and output parameters are relative to the Sham and active rhTMS protocols were dictated in real-time through an automated routine implemented in MatLab, and a TriggerBox device (EMS medical) connecting the operating PC with the TMS machine.

### 2.6. Data Analyses 

#### 2.6.1. EEG Data Preprocessing and Individual Alpha Frequency (IAF) Peaks Extraction

Preprocessing of rsEEG data was performed via EEGLAB [76]. Signals from every channel were offline and re-referenced to the average activity of all electrodes. Next, linear filtering was applied through a high-band pass of 1 Hz and a low-band pass of 40 Hz, which allowed us to minimize power line noise and motor artifacts. Filtered data underwent a further visual inspection, where temporal segments containing residual noise at occipital channels were rejected and spherically interpolated. 

After these preliminary quality checks, a power spectral density (PSD) estimate was performed, per each individual, on filtered data recorded from electrode Oz using Welch’s method (*pwelch* MatLab function). Resting-state signals were parsed into 2 s segments with a 25% degree of overlap. To reduce spectral leakage, a Hann-window was implemented on each segment, which was zero-padded to a length of 10 s to increase the frequency resolution up to 0.1 Hz. Each segment’s power spectrum was then computed via Fast Fourier Transform and averaged together with power spectra relative to all other segments. This procedure yielded the PSD estimate at each frequency bin and was reiterated for the Oz rsEEG data of every participant. 

Lastly, a custom-made MatLab algorithm was implemented to extract the IAF from the PSD estimate computed over rsEEG signals. This routine was designed to search for the frequency component within the narrowband spanning from 8 to 13 Hz that showed the highest power estimate. The output of this computation was double-checked by visually inspecting the PSD plot to ensure the estimated peak was representing a deviation from the 1/f scaling of the EEG spectral activity.

#### 2.6.2. Behavioral Data

Behavioral measurement kept track of performance accuracy (i.e., the inverse proneness to the SIFI: the higher the accuracy rates, the lower the propensity to experience the illusion), which was computed separately per each experimental block (i.e., each stimulation condition: Baseline, SHAM_NO, SHAM_O, TMS_NO, and TMS_O) as a function of the SOA (50, 100, and 150 ms). As one flash only was presented in every trial, accuracy rates were thus calculated as the ratio between the amount of correct responses (1-flash reports, being 2-flashes reports illusory in nature) over the total amount of trials. Such procedure yielded 15 distinct accuracy rates (one per each SOAs and stimulation condition), which were then entered into the statistical analyses.

### 2.7. Statistical Analyses 

All statistical analyses were performed via jamovi 2.3.21 (the jamovi project, 2021) and JASP 0.16.4 (the JASP team, 2022). We first approached our behavioral data by preliminarily analyzing accuracy rates at Baseline. This was done to ascertain whether the SIFI actually occurred at the group level (i.e., decreasing proclivity to report an illusory percept as the SOAs increased). To this aim, a repeated measure ANOVA (rmANOVA), having one within-subjects factor with three levels (corresponding to the three SOAs), was ran on mean accuracy rates. 

After, in order to address the main questions upon which the rationale of the current study rests, a further rmANOVA was performed. This analysis was designed to have stimulation condition (Baseline, SHAM_NO, SHAM_O, TMS_NO, and TMS_O) and SOAs (50, 100, and 150 ms) as within-subjects factors and mean accuracy rates as the dependent variable. This course of action enabled us to clarify whether rhTMS might be a convenient tool to investigate SIFI-related mechanics by testing, for example whether the entrainment protocol set at IAF modulates, as compared to Baseline, the illusion or not (providing a first causal demonstration against vs. in favor to the sampling rate hypothesis); or rather, disrupts the illusory phenomenon, due to any by-product thereof (clicking noise, cutaneous sensations). 

As the key scope of this study was to detail whether (and how) any of the stimulation protocols might differentially disrupt the occurrence of the illusion, the results yielded by this 5 × 3 rmANOVA were further explored via Bayesian Informative Hypotheses (BAIN). Compared to null-hypothesis testing, this approach allows us to directly evaluate how well a statistical model (built upon a hypothesis of interest) explains the experimental data. This could be achieved through the Bayes theorem by computing, per each hypothesis, the posterior model probability (PMP). Such a parameter, whose values span from 0 to 1, returns an estimate about the extent to which the associated model (as compared to a set of competing hypotheses) finds support from the empirical data. Accordingly, the higher the PMP, the higher the probability that the underlying hypothesis might be the best suited to describe the experimental evidence. Moreover, BAIN provides the possibility to contrast two distinct models (i.e., two distinct hypotheses) by comparing their relative PMPs and assessing whether one of them, relative to the other, has a higher probability of explaining the data [77,78]. 

In light of this statistical pipeline, a preliminary analysis performed on G*Power (parameters: effect size f = 0.25; α error probability = 0.05; power = 0.80; the number of groups = 1; the number of measurements = 15) returned an optimal sample size of 21 subjects for this kind of experimental design, which brought us to recruit 27 volunteers. 

## 3. Results

### Accuracy Rates 

**Figure 3:**
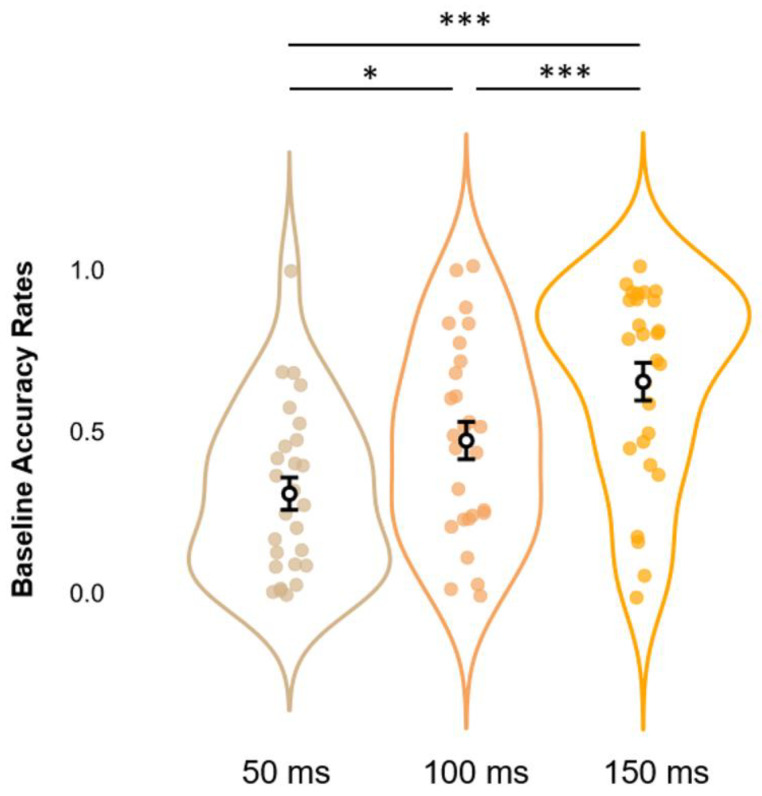
Estimated marginal means (error bars expressed as 95% standard errors) and violin plots relative to Baseline Accuracy Rates. The graph shows the observed Accuracy Rates scores (x-axis), sorted as a function of each different SOA (50, 100, and 150 ms; y-axis). * = *p* < 0.05, *** = *p* < 0.001.

Before moving to the core analyses, we first checked whether our data replicated findings regarding the SIFI by running on accuracy rates at Baseline a one-way rmANOVA with SOAs (3 levels: 50, 100, and 150 ms) as a within-subjects factor. Results show a significant main effect of SOAs (*F_2,52_ = 22.6, p < 0.001, η^2^_p_ = 0.465*)*,* which was further elucidated via post hoc analyses using Bonferroni corrections. This procedure confirmed the occurrence of the SIFI at the group level, as accuracy rates at 150 ms SOA (*M = 0.653, S.E.M. = 0.058, 95% C.I. = [0.553 0.773]*) proved to be significantly higher (all *p-values < 0.001*) than those relative to SOAs of 100 ms (*M = 0.471, S.E.M. = 0.058, 95% C.I. = [0.352 0.590]*) and 50 ms (*M = 0.307, S.E.M. = 0.050, C.I. = [0.204 0.410]*). Likewise, accuracy rates at 100 ms SOAs displayed significantly higher values as compared to those at 50 ms (*p-value < 0.05*). These data replicate findings relative to the differential impact that SOAs exert on the illusion, with larger SOAs decreasing the likelihood of perceiving a second, illusory flash (results depicted in Figure 3). 

We next performed our main analysis on accuracy rates to clarify whether rhythmic stimulation at occipital loci, delivered via Sham and active protocols, differentially affected or not the proneness to the SIFI. To this end, a 3 × 5 rmANOVA with SOAs (3 levels: 50, 100, and 150 ms) and Condition (5 levels: Baseline, SHAM_NO, SHAM_O, TMS_NO, and TMS_O) as within-subjects factors, and mean accuracy rates as dependent variable, was performed. Whereas a significant and robust main effect of SOAs was returned (*F_2,52_ = 34.13, p < 0.001, η^2^p = 0.568*), the analysis did not yield any main effect of Condition, nor any significant interaction of SOAs*Condition (all *F-values*_(*4,104*)(*8,208*)_ *< 2.31*, all *p-values > 0.05*, all *η^2^p < 0.081*).

**Figure 4:**
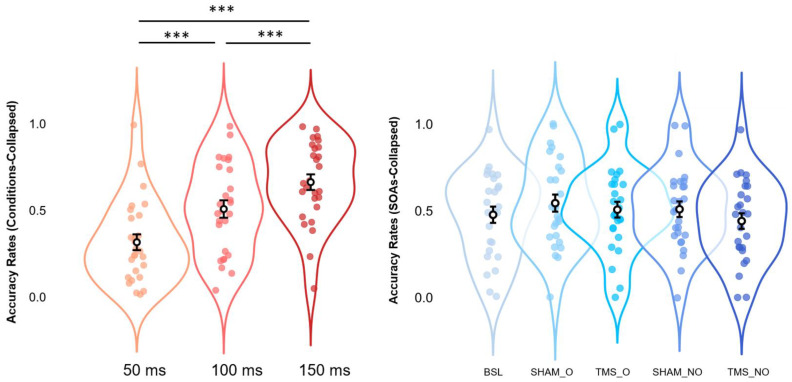
Estimated marginal means (error bars expressed as 95% standard errors) and violin plots relative to Condition-collapsed Accuracy Rates (leftmost panel) and SOAs-collapsed Accuracy Rates (rightmost panel). All graphs show observed Accuracy Rates scores (x-axes), sorted as a function of each different SOA (50, 100, and 150 ms; y-axes relative to the leftmost panel) or stimulation condition (BSL, SHAM_O, TMS_O, SHAM_NO, and TMS_NO; y-axis relative to the rightmost panel). *** = *p* < 0.001.

Post hoc analyses via Bonferroni corrections confirmed the effects observed in the Baseline condition alone. Independently of Condition, we compared within-subjects levels relative to the significant main effect of SOAs. Results suggest a pattern where Condition-collapsed accuracy rates at 150 ms (*M = 0.663, S.E.M. = 0.045, 95% C.I. = [0.570 0.755]*) appear to be higher (all *p-values < 0.001*) than those at 100ms (M = 0.508, S.E.M. = 0.050, 95% C.I. = [0.404 0.611]), and 50 ms (*M = 0.316, S.E.M. = 0.046, 95% C.I. = [0.222 0.410]*). In the same vein, Condition-collapsed accuracy rates reported for 100ms SOAs were shown to be significantly higher (*p-value < 0.001*) as compared to those at 50ms SOAs. All the above-mentioned results are depicted in Figure 4.

In light of the null findings revealed by the 5 × 3 rmANOVA regarding the effects of the stimulation protocols on the occurrence of the illusion, we implemented BAIN to better detail whether the equality-constrained hypothesis (i.e., similar mean accuracy rates across stimulation conditions) indeed retains the highest probability to describe our data. As such, we developed six different hypotheses about mean accuracy rates, and the PMPs thereof were then tested against each other. The first hypothesis (i.e., the one which we were focusing on) followed the results returned by the 5 × 3 rmANOVA, assuming that no significant difference in terms of accuracy rates (i.e., inverse proneness to the illusion) should be witnessed across conditions:

**H_1_**:  *Baseline = SHAM_NO = SHAM_O = TMS_NO = TMS_O*

The second hypothesis was a broad one and posited that, while no difference should be expected between active and Sham stimulation conditions (and between overlapping and non-overlapping stimulation protocols), the associated accuracy rates might be higher (i.e., reduced proneness to the illusion) than those relative to Baseline:

**H_2_**:  *Baseline < SHAM_NO = SHAM_O = TMS_NO = TMS_O*

The third and fourth hypotheses trailed the second one, as they assume in a more specific fashion that an increase in mean accuracy rates (as compared to Baseline) might be at work, depending on whether Sham or active TMS was delivered:

**H_3_**:  *Baseline < SHAM_NO = SHAM_O < TMS_NO = TMS_O*

**H_4_**:  *Baseline < TMS_NO = TMS_O < SHAM_NO = SHAM_O*

Likewise, the fifth and the sixth hypotheses posited that an increase in mean accuracy rates (as compared to Baseline) should be expected as a function of whether the stimulation protocols, regardless of their active or Sham nature, were administered so as to overlap or not with the occurrence of the first audiovisual pair:

**H_5_**:  *Baseline < SHAM_NO = TMS_NO < SHAM_O = TMS_O*

**H_6_**:  *Baseline < SHAM_O = TMS_O < SHAM_NO = TMS_NO*

**Figure 5:**
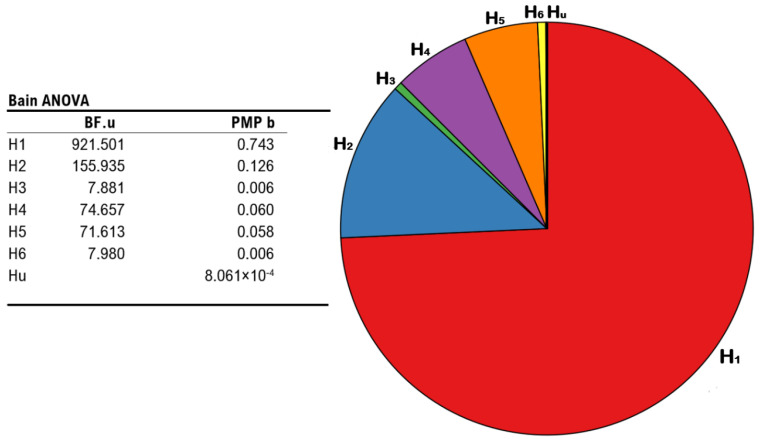
Results from BAIN regarding the impact of different stimulation protocols on the occurrence of the illusion. Leftmost panel: summary table showing Bayes Factors of each hypothesis vs. the unconstrained hypothesis (BF.u), and posterior model probabilities of each model (PMP b), including the unconstrained hypothesis. Rightmost panel: pie chart of the posterior model probabilities relative to all six models (H_1_, H_2_, H_3_, H_4_, H_5_, and H_6_), including the unconstrained hypothesis (H_u_).

Results show that H_1_ retained the highest relative probability (*PMPb = 0.743*), as compared to H_2_ (*PMPb = 0.126*), H_4_ (*PMPb = 0.060*), H_5_ (*PMPb = 0.058*), H_3_ and H_6_ (both *PMPb* values *= 0.006*), and Hu (*PMPb = 0.000*), which corresponds to unconstrained hypothesis representing any other set of relationships between the parameters other than those explicitly formulated (i.e., a “fail-safe” model that place no constraints on the parameters and mitigate the risk of selecting a set of bad hypotheses [77,79]). Accordingly, H_1_ also displays the highest Bayes Factor (*BF.u = 921.501*), as compared to all other hypotheses hereby outlined (all *BF.u* values *< 155.935*). Namely, when contrasting each of our hypotheses with H_u_, H_1_ corresponds to the model under which it appears more likely to report the patterns observed in our data, or else, the model receives the strongest support from our experimental evidence. All the above mentions results are depicted in Figure 5.

## 4. Discussion

In this study, we aimed to elucidate whether rhTMS may stand as a reliable tool with the potential to probe the cortical mechanics underlying the SIFI. In this regard, we investigated to which extent TMS-induced clicking noise and cutaneous sensations might perturb the occurrence of the illusion, as compared to Baseline. We addressed these experimental questions by delivering trains of interleaved magnetic pulses over the occipital pole during the pre-stimulus time window at a frequency rate matching the IAF of individuals asked to perform the SIFI task. This was done with regard to both Sham and active rhTMS following two distinct protocols, such that the last pulse within each train could either overlap or not with the occurrence of the audiovisual pair. Mean accuracy rates relative to each stimulation condition were tracked as behavioral proxies of the differential impact one of the stimulation conditions might exert on the SIFI.

To begin with, wide interindividual variability appears to subsume the SIFI. In our sample, we witnessed both illusion-prone participants (i.e., displaying accuracy rates close to 0 across different SOAs), and illusion-immune participants (i.e., displaying accuracy rates close to 1 across different SOAs). These data highlight how subjective in nature the SIFI is and the degree to which the associated phenomenal outcomes might change as a function of individual factors [17]. Some of them have been linked to training conditions [80], pathological aging [81], and, most of all, clinical and subclinical neuropsychiatric conditions, such as autism spectrum disorder [8], schizophrenia [58], and marked schizotypal traits [57]. At the empirical level, these two latter cases epitomize a biobehavioral continuum along which alterations within the perceptual (and phenomenal) domain qualitatively change depending on how severe the syndromic landscape is [11,82]. Illusions entail misperceiving one or more properties of an actual object, whereas no such object exists at all (even though reported) when hallucinatory episodes occur [83]. Accordingly, proneness to illusions, such as the SIFI, was found to be increased in both schizophrenic patients and individuals with high schizotypal traits [57,58], whilst hallucinations are a syndromic hallmark of schizophrenia only. In brief, the worse the clinical scenario, the broader the phenomenal alterations. These pieces of evidence emphasize the translational meaning of the SIFI as a reliable index of multisensory deficits, which should be capitalized on to illuminate the putative pathomechanisms arbitrating the individual’s trajectory toward a neuropsychiatric phenotype or another.

Away from these preliminary considerations, our analyses yielded rather intriguing results. First, we replicated findings concerning the reliance of the SIFI on different SOAs. Data collected at Baseline confirmed a higher propensity to double-flash reports (i.e., lower accuracy rates) for inter-beep delays around 50 ms [15], with progressively increasing accuracy rates (i.e., lower proneness to the SIFI) as the SOAs got wider [42,57]. The larger the inter-beep delays, the easier and more accurate the cross-modal integration.

Such findings provided a convenient departure point toward an in-depth inquiry about the impact different stimulation conditions may exert on the SIFI, as compared to Baseline. According to our results, none of the TMS protocols significantly perturbed the illusory rate. On the one hand, we delivered the Sham stimulation to assess whether TMS clicks might interfere with the ongoing perceptual processing of acoustic stimuli. We found no evidence for either an enhancement or suppression of the double-flash report across each SOA, as compared to Baseline. Likewise, active rhTMS was administered to test for unintended changes to the illusory experience due to somatosensory sensations that come along with the stimulation. No such evidence was found either. In addition, this allowed to discount for a perceptual bias effect potentially induced by the rhTMS delivered precisely at IAF as causative for the illusory rate report, i.e., an alternative account to the sampling rate hypothesis (see [41]). Both the Sham and active protocols were also set to either overlap or not with the occurrence of the audiovisual pair. As the former condition was proven to instantiate the maximal oscillatory entrainment [38,60,61], we aimed to ascertain it did not prompt any differential alteration to the SIFI, as compared to the non-overlapping stimulation. Again, no difference between conditions could be found. These patterns were further corroborated via Bayesian statistics, which confirmed that the best-suited model to explain our observed data was indeed one where none of the stimulation protocols had an impact on the occurrence of the illusion. Taken together, these findings provide novel evidence about the feasibility of rhTMS-based interventions in the cortico-rhythmic scrutiny of audiovisual integration. That is, the experimental by-products engendered by rhTMS (i.e., clicking noise and somatosensations) do not jeopardize the occurrence of the illusion, whose cortical underpinnings could be thus further explored by means of this protocol. This is of uttermost significance in translational terms, as it implies that rhTMS could be capitalized on (in forthcoming studies) to gather clinical insights into the electrocortical signatures driving multisensory impairments witnessed in psychosis-prone individuals. Such considerations hold true, especially with regard to the combination of rhTMS and EEG recordings.

Although EEG and neuromodulation have been separately exploited to this end [42,45], no attempt to combine these techniques has been carried out until now due to tACS-induced artifacts contaminating EEG signals [53]. The findings herein supplied pave instead the way to forthcoming inquiries in which both behavioral and electrocortical insights into the SIFI could be gained. Indeed, TMS (as compared to tACS) retains some favorable biophysical properties that ushered in the development of pipelines aimed at denoising EEG recordings from TMS-induced artifacts [84,85,86]. As such, TMS-EEG has progressively become a reliable approach to address what more oscillations can tell us about neural dynamics and their psychological correlates [87,88]. TMS-EEG co-registration has so far been employed to explore the hodological architecture of the cortical connectome [89] and whether any causal interplay between distinct neural nodes therein may occur under specific physiological or cognitive conditions [90]. Despite being focused on the response of local rhythms to an external oscillator, online EEG matched with rhTMS fall within this framework as well [38,60,67,68]. When conflated with our results, these notions streamline the implementation of combined rhTMS-EEG to test for the oscillatory underpinnings of the SIFI. This even more so applies if the clinical significance of specific alpha features (i.e., IAF) to specific psychiatric disorders is factored in [91].

Aside from these considerations, our main analyses are also suggestive of an even stronger impact of SOAs on the likelihood of experiencing the illusion. Akin to what has been outlined at Baseline, wider inter-beep delays (as compared to narrower ones) come with a lesser proclivity to double-flash reports [15,17,42]. This evidence remarks the robustness of the SIFI and the extent to which this phenomenon is reliant on the cross-modal interplay between audition-relayed priors and occipital excitability [23,25,92]. The more the former is conveyed within a temporal frame outpacing the spontaneous excitatory cycles (i.e., TBWs) of the visual cortex, the worse the multisensory operations are instantiated.

Germane to this notion, it could be argued that active rhTMS tailored at the IAF should have prompted an increase in alpha power and a reduction in cortical excitability, which might have, in turn modulated the probability of reporting the illusion [27,28,42]. Our data provide no evidence in support of such a claim. These results might be construed as borrowing distinct empirical and theoretical perspectives. For instance, the inverse relationship between the amplitude of alpha rhythms and the proneness to the illusion across SOAs was reported in one study only [42] and has barely replicated ever since. Furthermore, the tie between transient fluctuations in posterior alpha power and visual performance has been recently couched within a broader conceptual framework accounting for signal detection theory (SDT) measurements rather than accuracy per se [93]. While perceptual sensitivity (i.e., d-prime) should be dictated by the cycling speed of occipital alpha waves [38], amplitude bursts in the same frequency band are assumed to channel sensory biases (i.e., response criterion) [94], and shape metacognitive abilities [95]. As our experimental design was primarily planned to measure the feasibility of the application of the rhTMS protocol in testing the neural underpinnings of the SIFI without altering its phenomenological experience across individuals, it does not allow us, in its current form, to extract SDT-derived indices. Therefore, it remains an open question whether rhTMS modulation of either alpha power or frequency may causally impact the observer’s sensory bias vs. perceptual sensitivity when engaged in multisensory paradigms, such as the SIFI. Nevertheless, we have provided convincing evidence here that this protocol is viable to causally test for the functional role of different oscillatory parameters in determining the SIFI. Moreover, although we could not disentangle with the current paradigm whether any modulation in the proneness to the illusion could be determined by a perceptual bias or change in perceptual sensitivity, the lack of any modulation by the active compared to the Sham stimulation speaks in favor of the absence of a perceptual bias phenomenon as far as the entrainment at IAF is concerned. Indeed, according to the sampling rate hypothesis, this effect should be expected for stimulation at a faster or slower pace than IAF, a hypothesis that can be causally tested using the protocol hereby validated. Such reasoning has some beneficial implications concerning the functional scrutiny of cortical rhythms unfolding outside the canonical boundaries of the alpha band as well. That is, rhTMS-driven entrainment could be deployed not only to up- or down-shift the alpha peak (i.e., IAF ± 1 or 2 Hz [38,42]) but also to recruit neuronal pools oscillating, for instance, within the theta (i.e., around 5 Hz) or beta (i.e., around 20 Hz) range. Akin to what aforementioned about alpha activity, this would allow us to causally probe how pivotal the contribution of these rhythms is to cross-modal operations, trailing empirical findings recently gathered (from occipital sensors) by means of both audio-visual and visuo-tactile paradigms [71,72,96,97].

To conclude, we herein replicated results regarding the robustness of the SIFI as an illusory event and the degree to which it relies on interindividual variability and distinct temporal dynamics (i.e., SOAs). Moreover, we also provided evidence for the viability of rhTMS-based protocols to explore this phenomenon. Namely, TMS-induced clicks and somatosensations do not significantly alter the occurrence of the illusion. These findings lay the foundations for future inquiries set to question (by means of rhTMS-EEG co-registration) the causal contribution of specific electrocortical features (i.e., alpha, and theta and beta frequency and amplitude) to perceptual metrics of cross-modal integration such as visual acuity and response bias. 

## Data Availability

The data presented in this study and the Matlab code for data analysis are available at the following link (OSF link will be made available upon acceptance of the manuscript).
